# Does hand modulate the reshaping of the attentional system during rightward prism adaptation? An fMRI study

**DOI:** 10.3389/fpsyg.2022.909815

**Published:** 2022-07-27

**Authors:** Nicolas Farron, Stephanie Clarke, Sonia Crottaz-Herbette

**Affiliations:** Neuropsychology and Neurorehabilitation Service, Centre Hospitalier Universitaire Vaudois (CHUV), University of Lausanne, Lausanne, Switzerland

**Keywords:** prism adaptation, functional reshaping, attention, inferior parietal lobule, hand, fMRI

## Abstract

Adaptation to right-deviating prisms (R-PA), that is, learning to point with the right hand to targets perceived through prisms, has been shown to change spatial topography within the inferior parietal lobule (IPL) by increasing responses to left, central, and right targets on the left hemisphere and decreasing responses to right and central targets on the right hemisphere. As pointed out previously, this corresponds to a switch of the dominance of the ventral attentional network from the right to the left hemisphere. Since the encoding of hand movements in pointing paradigms is side-dependent, the choice of right *vs*. left hand for pointing during R-PA may influence the visuomotor adaptation process and hence the reshaping of the attentional system. We have tested this hypothesis in normal subjects by comparing activation patterns to visual targets in left, central, and right fields elicited before and after adaptation to rightward-deviating prisms using the right hand (RWRH) with those in two control groups. The first control group underwent adaptation to rightward-deviating prisms using the *left* hand, whereas the second control group underwent adaptation to *leftward*-deviating prisms using the right hand. The present study confirmed the previously described enhancement of left and central visual field representation within left IPL following R-PA. It further showed that the use of right *vs*. left hand during adaptation modulates this enhancement in some but not all parts of the left IPL. Interestingly, in some clusters identified in this study, L-PA with right hand mimics partially the effect of R-PA by enhancing activation elicited by left stimuli in the left IPL and by decreasing activation elicited by right stimuli in the right IPL. Thus, the use of right *vs*. left hand modulates the R-PA-induced reshaping of the ventral attentional system. Whether the choice of hand during R-PA affects also the reshaping of the dorsal attentional system remains to be determined as well as possible clinical applications of this approach. Depending on the patients' conditions, using the right or the left hand during PA might potentiate the beneficial effects of this intervention.

## Introduction

In the wake of the seminal report on alleviation of neglect syndromes by brief exposure to rightward-deviating prisms (Rossetti et al., 1998), numerous studies investigated sensorimotor and cognitive effects in patients with neglect and normal subjects (for review see e.g., Michel, 2016). Prism adaptation with rightward deviating prisms (R-PA) was shown to yield consistent sensori-motor and cognitive effects in patients with right hemispheric lesions and sensori-motor after-effects in normal subjects but only rarely cognitive effects in the latter (Colent et al., 2000). The paucity of visuo-spatial and other cognitive effects in normal subjects has been repeatedly pointed out (e.g., Fortis et al., 2011; Schintu et al., 2014, 2017).

There are three very specific instances, in which behavioral effects of R-PA were reported in normal subjects. First, R-PA was shown to speed up reflexive re-orienting from invalid cues on the left to targets on the right side, when the Posner paradigm was used, and this in subjects with an initial large cueing effect (Striemer et al., 2006). Second, R-PA was reported to induce a rightward shift in visual midpoint judgment in extrapersonal (but not peripersonal) space (Berberovic and Mattingley, 2003). Third, R-PA yielded greater effects in a subgroup of subjects, who had before prism exposure a lesser attentional bias in favor of the left side of space. The attentional bias for the left, referred to as pseudoneglect, is readily found in normal subjects who, in the line bisection task, tend to estimate the middle of a line to the left of the objective midpoint (Jewell and McCourt, 2000). Pseudoneglect is believed to reflect right hemispheric dominance for visuospatial processing (Fink et al., 2001; Corbetta et al., 2005). A recent study (Schintu et al., 2021) highlighted the role of the posterior parietal cortex inline bisection judgment and visuospatial bias. When the function of the right posterior parietal cortex was temporarily disrupted in normal subjects by theta burst TMS, the subjects presented a rightward shift in line bisection judgment, accompanied by increased resting state functional connectivity between the right posterior parietal cortex and the left superior temporal gyrus (Schintu et al., 2021). The authors stressed the role of structural interhemispheric connections on the basis of a correlation with fractional anisotropy within the posterior callosal pathway. The inter-individual variability in the extent of pseudoneglect accounts, at least partially, for differences in cognitive effects after R-PA, as larger pseudoneglect at baseline (i.e., before R-PA) is associated with greater effects (Goedert et al., 2010; Herlihey et al., 2012). Interestingly, the extent of pseudoneglect in line bisection depends on the hand used; it tends to be smaller when the right hand, as compared to the left hand, is used (Scarisbrick et al., 1987; Fukatsu et al., 1990; Brodie and Pettigrew, 1996; Jewell and McCourt, 2000).

The interaction between the effect of exposure to prisms and motricity is currently of great interest. Recent studies reported very promising results in the prolongation of PA effects; they showed that tDCS applied to the primary motor cortex strengthens PA-induced after-effects and boosts the therapeutic effect of R-PA in neglect (review: Panico et al., 2021).

R-PA modulates neural activity within regions involved in visuo-motor transformation, with a subsequent impact on the organization of the attentional system, as demonstrated in five fMRI studies. All five studies used the right hand for pointing during adaptation. An event-related fMRI study, carried out in normal subjects, reported, during pointing trials executed with the right hand, a transient increase of activity within the primary motor cortex, the anterior cingulate cortex, and the anterior part of the intraparietal sulcus on the left side (Danckert et al., 2008). The impact on the attentional system was investigated in normal subjects in two fMRI studies, which used paradigms known to involve the ventral attentional network (VAN) and compared patterns of activation before *vs*. after R-PA. The first study used visual targets and reported an increase in activation by left, central, and right targets in left IPL and a decrease in activation by right and central targets in right IPL (Crottaz-Herbette et al., 2014). The second study used auditory targets and demonstrated an increase in activation by left, central, and right auditory targets in left IPL and a decrease in activation by right auditory targets in right IPL (Tissieres et al., 2018). These results were interpreted as a switch in dominance of VAN from the right to the left hemisphere (Clarke and Crottaz-Herbette, 2016).

The impact of R-PA on activation patterns elicited by attentional tasks has also been investigated in patients with right-hemispheric lesions. The task of visual target detection revealed an enhancement of the representation of the left and central visual fields in the left hemisphere (Crottaz-Herbette et al., 2017a). Line bisection and visual search, known to involve the dorsal attentional network (DAN), demonstrated an increase in activation within the superior parietal lobule, the superior frontal gyrus, and the lateral occipital cortex on the left side and, when spared, on the right side (Saj et al., 2013).

As pointed out in a seminal review, visuomotor recalibration, that is, the correction of pointing errors early in R-PA, involves the superior parietal cortex and is the starting point of brain reorganization, which spreads to other regions and affects several cognitive domains (Panico et al., 2020). The superior parietal cortex is known to be involved in visuo-motor control, including reaching movements (Connolly et al., 2003; Culham et al., 2006). Pointing, in particular, depends on the intraparietal sulcus in either hemisphere; neural activity within the intraparietal sulcus is modulated by the position of the target (stronger for contra- than ipsilateral targets) and by the respective positions of target and hand (stronger for contralateral hand reaching for contra- than ipsilateral targets (Medendorp et al., 2005).

On the basis of the above evidence of side-dependent encoding of hand movements in pointing paradigms (Medendorp et al., 2005), we formulated our working hypothesis as follows. The choice of right *vs*. left hand for pointing during R-PA influences the recalibration and spatial realignment process and hence the reshaping of the attentional system. We have tested this hypothesis with respect to the previously reported re-organization of VAN (Crottaz-Herbette et al., 2019) by comparing activation patterns to visual targets in left, central, and right fields elicited before and after adaptation to rightward deviating prisms using the right hand (RWRH) with those in two control groups. The first control group underwent adaptation to rightward-deviating prisms using the *left* hand (RWLH), whereas the second group underwent adaptation to *leftward*-deviating prisms using the right hand (LWRH).

## Materials and methods

### Participants

This experiment included 36 healthy adults, who were separated into 3 groups. One group used rightward deviating prisms and their right hand during the adaptation (RWRH group, 14 participants, 5 men, 26.0 ± 4.9 years). A second group used rightward deviating prisms and their left hand during the adaptation (RWLH group, 11 participants, 6 men, 24.9 ± 2.9 years). A third group used leftward deviating prisms and their right hand during the adaptation (LWRH group, 11 participants, 4 men, 22.0 ± 1.7 years). All participants were right-handed, had normal or corrected vision, had no major psychiatric or neurological illnesses. All participants gave written informed consent according to procedures approved by the Ethics Committee of the Canton de Vaud.

### Procedure

All participants followed the same procedure. PA was preceded and followed by fMRI acquisitions during a visual detection task. PA was done outside the scanner.

### Visual detection task

During fMRI acquisitions, all participants did a simple visual detection task as described in previous studies (Crottaz-Herbette et al., 2014, 2017b). Briefly, subjects were asked to respond as quickly as possible, by pressing a response button with the forefinger, when the visual target stimulus appeared (a white star on a black background). Targets appeared for 500 ms in the midsagittal plane (0°), 20° on the left, or 20° on the right in a pseudorandom order. The three locations were equally used, 20 times in each location. The inter-stimulus interval varied between 1 and 20 s. The RWRH and LWRH groups used the right hand to press the button during this task and the RWLH group used the left hand. The task was programmed using the software E-Prime 2.0 (Psychology Software Tools https://pstnet.com/products/e-prime/). The duration of the task was 6 min 44 s. The reaction time and the accuracy for each target were recorded.

The same experimental set-up with central fixation has been used in a previous study, which compared R-PA-induced modulation on three visual tasks: (i) visual detection (as in this study); (ii) visuospatial short-term memory; and (iii) verbal short-term memory (Crottaz-Herbette et al., 2014). Changes in activation patterns before *vs*. after a brief exposure to R-PA, as compared to plain glasses, were driven by a selective modulation during the visual detection task. Visual targets, similar to the ones used in this study, were large bright white stars on a black background and were easily detected without eye movements.

### Visuomotor adaptation

The prism adaptation was performed outside the scanner using 10° rightward deviating lenses for the RWRH and RWLH groups and 10° leftward deviating lenses for the LWRH group prisms (www.optiquepeter.com). A deviation of 10° was chosen as (Facchin et al., 2013) have shown in their previous study that prisms with this deviation magnitude (corresponding to 20 prismatic dioptre) induced large and robust after-effect in an open-loop task. In addition, using a deviation of 10° allows comparing the findings of the present study with those observed in our previous studies (Crottaz-Herbette et al., 2014, 2017b, 2019; Tissieres et al., 2017). It also provides insights that are also pertinent for patients as 10° is the magnitude the most often used in patients and using a larger magnitude of deviation would have influenced the after-effects, and probably, the subsequent brain modulation.

PA consisted of approximately 150 pointing (3 min) with the right (RWRH and LWRH groups) or the left (RWLH group) forefinger to visual targets presented 14° to the left or the right of the midsagittal plane. The head of participants was immobilized in a headrest, and two-thirds of the pointing trajectories were hidden from their view. After a few numbers of trials showing initial error in the direction of the prisms deviation, all participants pointed correctly to the targets. The after-effect was assessed before and immediately after the prisms were removed; the participants were asked to look (without the prisms) at the visual target, then close their eyes and reach for the visual target with the index finger used during the PA. For each subject and each target position, we put a mark on the table where the participant pointed and we measured the deviation between the pointing and the actual target. The same procedure was repeated two times for the left target and two times for the right target in random order. For each side separately, we averaged together the two pointings. By convention, positive values represent a deviation to the right of the targets and negative values a deviation to the left of the targets. To compare the amount of after-effect between groups, these means were converted into absolute values and the difference between the pre- and post-PA measures were analyzed with an ANOVA including the between-subjects factor Group (RWRH, RWLH, LWRH) and the within-subjects factor Target side (left, right). *Post-hoc* analyses were further conducted when significant effects were obtained in the ANOVA.

### Data acquisition

Event-related fMRI and structural MRI data acquisition were conducted at the Lemanic Biomedical Imaging Center (Center d'Imagerie Biomédicale) in the Center Hospitalier Universitaire Vaudois, Lausanne on a 3T Siemens Magnetom Trio scanner with a 32-channel head-coil for the RWRH group, and a 3T Siemens Prisma scanner with a 64-channel head-coil for the RWLH and LWRH groups. Movements of the participants' head were prevented by adding padding around each participant's head in the coil. fMRI acquisitions used a single-shot echo-planar imaging gradient echo sequence (repetition time: 2 s; flip angle: 90°; echo time: 30 ms; number of slices: 32; voxel size: 3 x 3 x 3 mm; 10% gap). In total, 32 slices covering the whole head volume were acquired in the AC–PC plane in sequential ascending order. A high-resolution T1-weighted 3D gradient-echo sequence (160 slices, voxel size 1 x 1 x 1 mm) was also acquired for each participant.

### Data analysis

Behavioral performance during the visual detection task was assessed with mixed-design ANOVA including the between-subjects factor Group (RWRH, RWLH, LWRH) and the within-subject factors Target location (left, center, right) and Session (pre, post). *Post-hoc* analyses were further conducted when significant effects were obtained in the ANOVA.

Neuroimaging data were processed using the software Statistical Parametric Mapping (SPM12, Wellcome Department of Cognitive Neurology, London, United Kingdom, http://www.fil.ion.ucl.ac.uk/ spm/software/spm12/). For functional data, motion correction was performed by applying a 6-parameter rigid-body transformation minimizing the difference between each image and the first scan. The participants' anatomic images were co-registered to the functional realigned images and then normalized to the Montreal Neurological Institute (MNI) template using a 12 parameters affine transformation. Finally, the realigned and normalized functional images were resliced to obtain a 2 x 2 x 2 mm voxel size and spatially smoothed using an isotropic Gaussian kernel of 6-mm FWHM to increase signal-to-noise ratio. The first level statistics for each participant used a general linear model, as implemented in SPM12 software. The realignment parameters were included in the model as regressors. For all participants, contrasts of interest were specified for each session. The second-level (group-level) statistics based on the random field theory used the maps generated from these individual contrasts of interest. All group analyses were restricted to voxels with the probability of belonging to gray matter >50%, as defined in the *a priori* template available in SPM.

Group analyses started with a general ANOVA using the between-subjects factor Group (RWRH, RWLH, LWRH), and the within-subjects factors Target location (left, center, right) and Session (pre, post). Then, to disentangle the brain changes related to the prism deviation from those related to the hand used during the adaptation, one ANOVA included the RWRH and RWLH groups (assessing the hand-related effects), and another ANOVA included the RWRH and LWRH groups (assessing the prisms deviations' effects). These two ANOVAs included the between-subjects factor Group (RWRH, RWLH or RWRH, LWRH), the within-subject factors Target location (left, center, right), and Session (pre, post). Surface rendering images were done using the software BrainNet Viewer http://www.nitrc.org/projects/bnv/) (Xia et al., 2013) and the toolbox bspmview (http://www.bobspunt.com/software/bspmview) using the statistical maps obtained from the interactions and main effects set at a threshold of *p* < 0.05 and to a cluster extent corresponding to the number of expected voxels per cluster determined by SPM.

Further measures of activation in 3-mm diameter spheres located in the main peaks of the left and right IPL in the three-way interaction of the general ANOVA were done using MarsBaR toolbox (https://marsbar-toolbox.github.io/) (Brett et al., 2002). The locations of these regions of interest (ROIs) are visualized in a glass brain with the BrainNet Viewer (http://www.nitrc.org/projects/bnv/) (Xia et al., 2013). For each group and each target position, differences between the post- minus pre-session were computed for each ROI and displayed in a matrix figure.

## Results

### Visuomotor after-effect

The pointing error, which was measured with open-loop pointing to the left and right targets immediately after the adaptation, was always in the opposite direction of prism deviation. The extent of visuomotor after-effect was compared between groups using absolute values (Table 1). A mixed-design ANOVA on the difference between the pre- and post-PA measures including between-subjects factor Group (RWRH, RWLH, LWRH) and within-subjects factor Target side (left, right) yielded significant main effects of factors Group (F_(2, 33)_ = 6.50, *p* = 0.0041) and Target side (F_(2, 33)_ = 9.07, *p* = 0.0049), whereas the interaction was not significant.

**Table 1:** Behavioral data for the RWRH, RWLH, and LWRH groups: averages and standard errors for the after-effect and the detection task.

**Groups**	**After-effect**		**Reaction time (ms)**						**Accuracy (%)**					
	**(mm)**		**Pre**			**Post**			**Pre**			**Post**		
	**Left**	**Right**	**Left**	**Center**	**Right**	**Left**	**Center**	**Right**	**Left**	**Center**	**Right**	**Left**	**Center**	**Right**
RWRH	66 ± 4	56 ± 5	399 ± 47	388 ± 44	401 ± 50	415 ± 71	394 ± 47	407 ± 51	98 ± 4	100 ± 0	97 ± 6	99 ± 2	99 ± 3	100 ± 1
RWLH	44 ± 9	26 ± 5	374 ± 52	352 ± 52	371 ± 53	372 ± 51	340 ± 50	369 ± 60	100 ± 0	100 ± 0	100 ± 0	100 ± 0	100 ± 0	100 ± 0
LWRH	51 ± 7	47 ± 4	365 ± 35	365 ± 42	366 ± 35	372 ± 8	347 ± 20	383 ± 36	100 ± 0	100 ± 0	100 ± 0	100 ± 0	100 ± 0	100 ± 0

After-effect (in mm), for left and right targets, measured outside the scanner, before and after PA in an open-loop pointing task. To compare the magnitude of the deviation, all measures are reported in absolute value. Reaction time (ms) and accuracy (%) for left, center, and right visual targets during the detection task, measured before (pre) and after (post) PA, during the fMRI acquisition.

The main effect of Target side was driven by larger after-effects for the left than the right targets (Table 1). When groups were analyzed separately, the after-effect was larger for left than right targets (Table 1).

The main effect of the Group was driven by a significant difference between the RWRH and RWLH groups (t_(24)_ =-3,61, *p* = 0.003), with a larger after-effect for the RWRH. The difference neither between the RWRH and LWRH groups nor between the RWLH and LWRH groups was significant.

### Behavioral results

All subjects performed the target detection task without difficulties. Accuracy was at or near ceiling level (Table 1). Thus, RA-induced modulation of activation patterns elicited by Target detection, which we report below, did not reflect change in performance at this task, but genuine changes in visuo-spatial representations. Reaction times were analyzed with a general mixed-design ANOVA with Group (RWRH, RWLH, LWRH) as between-subject factor and Target location (left, center, right) and Session (Pre, Post) as within-subjects factors, which yielded significant main effect of Target location (F_(2, 62)_ = 21.88; *p* < 0.001). No other main effect or interaction was significant. *Post-hoc* analysis showed that the reaction time for central targets was significantly faster than the reaction time for left (t_(24)_ =5,57 *p* < 0.0001) or right targets (t_(24)_ =-5,85 *p* < 0.0001); the difference between left and right targets was not significant.

### Intervention-related changes in activation patterns

**Figure 1:**
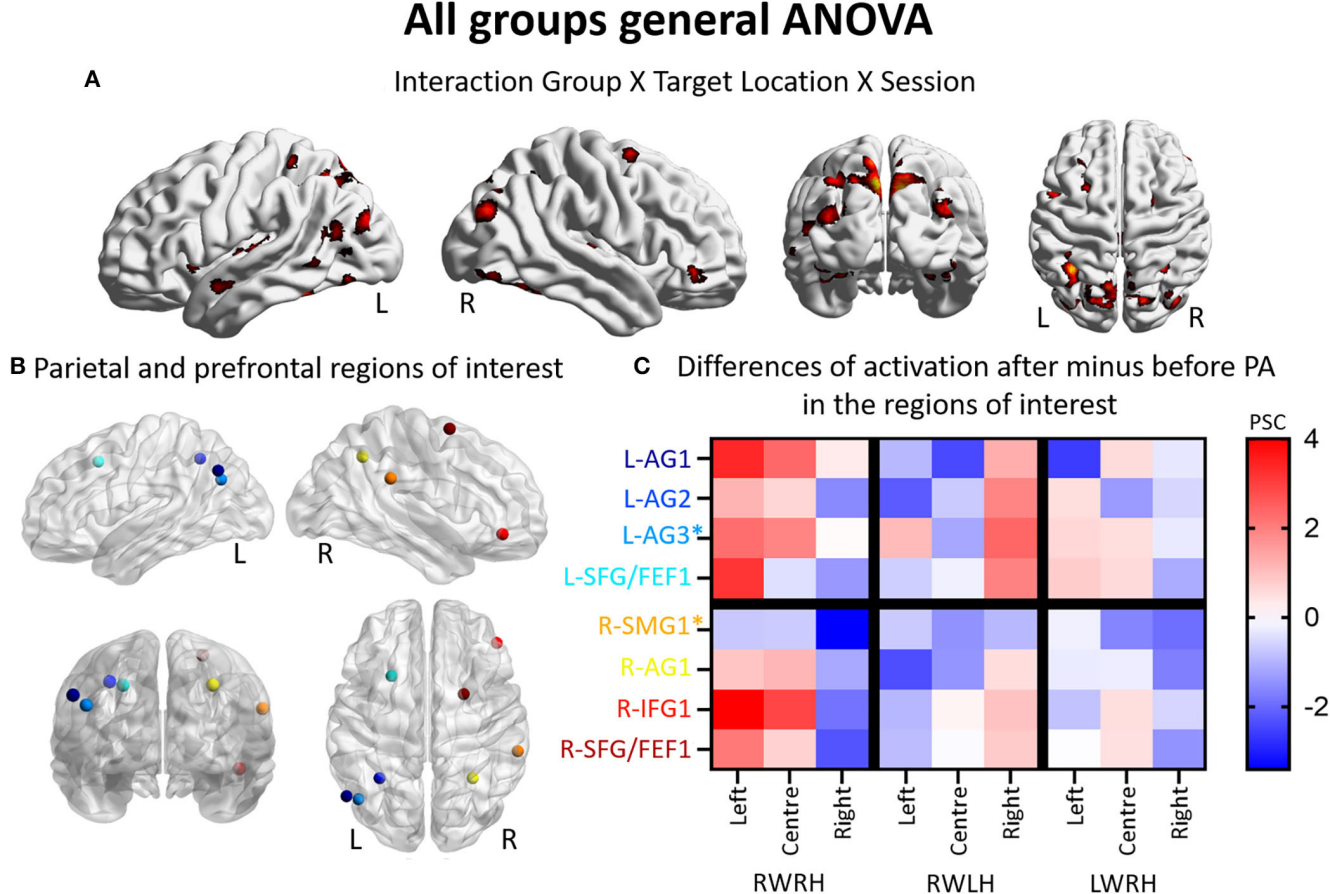
Modulation of brain activation as revealed by the general ANOVA including the three experimental groups (RWRH, RWLH, LWRH). **(A)** Surface renderings of significant brain activation in the interaction between the factors Group (RWRH, RWLH, LWRH) x Target location (left, center, right) x Session (pre, post), from left to right: lateral views of the left and right hemisphere, view from the back and the top. All maps are thresholded at *p* < 0.05, cluster extent k > 64. **(B)** Spheres located on the peaks of activation clusters in the parietal and prefrontal regions. Lateral views of the left and right hemispheres, above the back and top views. Color code denotes names of the regions, as indicated on the *y*-axis in C. **(C)** Differences of activation in percent signal change, after minus before PA, for each group (RWRH, RWLH, and LWRH) and each target position (left, center, and right). Asterisks mark two additional regions, which were identified in a previous study by contrasting the effect of R-PA to that of plain glasses (Crottaz-Herbette et al., 2014). Activation thresholded at *p* = 0,05 and k = 64 (expected numbers of voxels per clusters provided by SPM12) 0.4 AG, angular gyrus; FEF, frontal eye field; IFG, inferior frontal gyrus; L, left; PSC, percent signal change; R, right hemisphere; SFG, superior frontal gyrus; SMG, supramarginal gyrus.

Activation patterns elicited by the task of visual detection were modulated by prism adaptation. The intervention-related changes in activation patterns were investigated with a mixed-design ANOVA with Group (RWRH, RWLH, LWRH) as between-subject factor and Target location (left, center, right) and Session (Pre, Post) as within-subjects factors. The interaction Group x Target location x Session was significant in several clusters within the left and right hemispheres (Figure 1A, Table 2). In particular, the interaction was significant especially bilaterally in the IPL, superior parietal lobule, superior frontal gyrus, frontal eye field, middle occipital gyrus, precuneus; in the right inferior frontal gyrus; and in the left superior temporal gyrus (see Table 2 for a complete list of activations).

**Table 2:** Brain regions showing significant effects in the interaction between the factors group x session x target location, in the three-way ANOVA including the 3 groups (RWRH, RWLH, LWRH).

**3 Groups: RWRH RWLH LWRH**	**N of**	**Peak**	**Peak MNI** **coordinates**		
**Areas**	**voxels**	**intensity**	**x**	**y**	**z**
Left STG, MTG	125	7.22	−48	−2	−10
Bilateral precuneus, posterior cingulate gyrus, AG, cuneus, Left SPL, IPL, Right middle occipital gyrus (including L-AG2)	2,406	7.01	14	−66	38
Right parahippocampal gyrus, limbic lobe, STG, fusiform gyrus	66	6.87	30	−46	−14
Left fusiform gyrus, MTG, middle occipital gyrus, AG, STG (including L-AG1)	812	6.84	−40	−46	−16
Left caudate, anterior cingulate gyrus, limbic lobe	192	6.38	−14	22	−6
Left MFG, SFG (including L-SFG/FEF1)	261	5.48	−24	16	42
Left posterior cingulate gyrus, precuneus	161	5.07	−14	−38	40
Right IPL, AG (including R-AG1)	87	5.01	30	−52	42
Left STG, transverse temporal, precentral, and postcentral gyri	232	4.95	−48	−16	12
Right MFG, IFG (including R-IFG1)	77	4.74	46	38	−8
Right declive of vermis, cerebellum 6, vermis 7	121	4.73	12	−68	−26
Right precentral gyrus, STG	155	4.54	48	−16	12
Left posterior cingulate, parahippocampal, and lingual gyri	156	4.53	−14	−48	2
Left postcentral gyrus, IPL	88	4.49	−38	−36	66
Right parahippocampal gyrus, limbic lobe, amygdala	95	4.40	28	−8	−10
Left superior occipital gyrus, MTG, AG	186	4.34	−38	−82	26
Right MFG, SFG (including R-SFG/FEF1)	72	4.10	24	4	60
Left insula, IFG	68	3.98	−38	12	2
Right ITG, middle and inferior occipital gyri	291	3.96	48	−60	−12
Right postcentral gyrus, precuneus	96	3.93	8	−66	58
Left MTG, STG	74	3.86	−66	−34	4
Left IFG, MFG	147	3.77	−48	6	28

**Figure 2:**
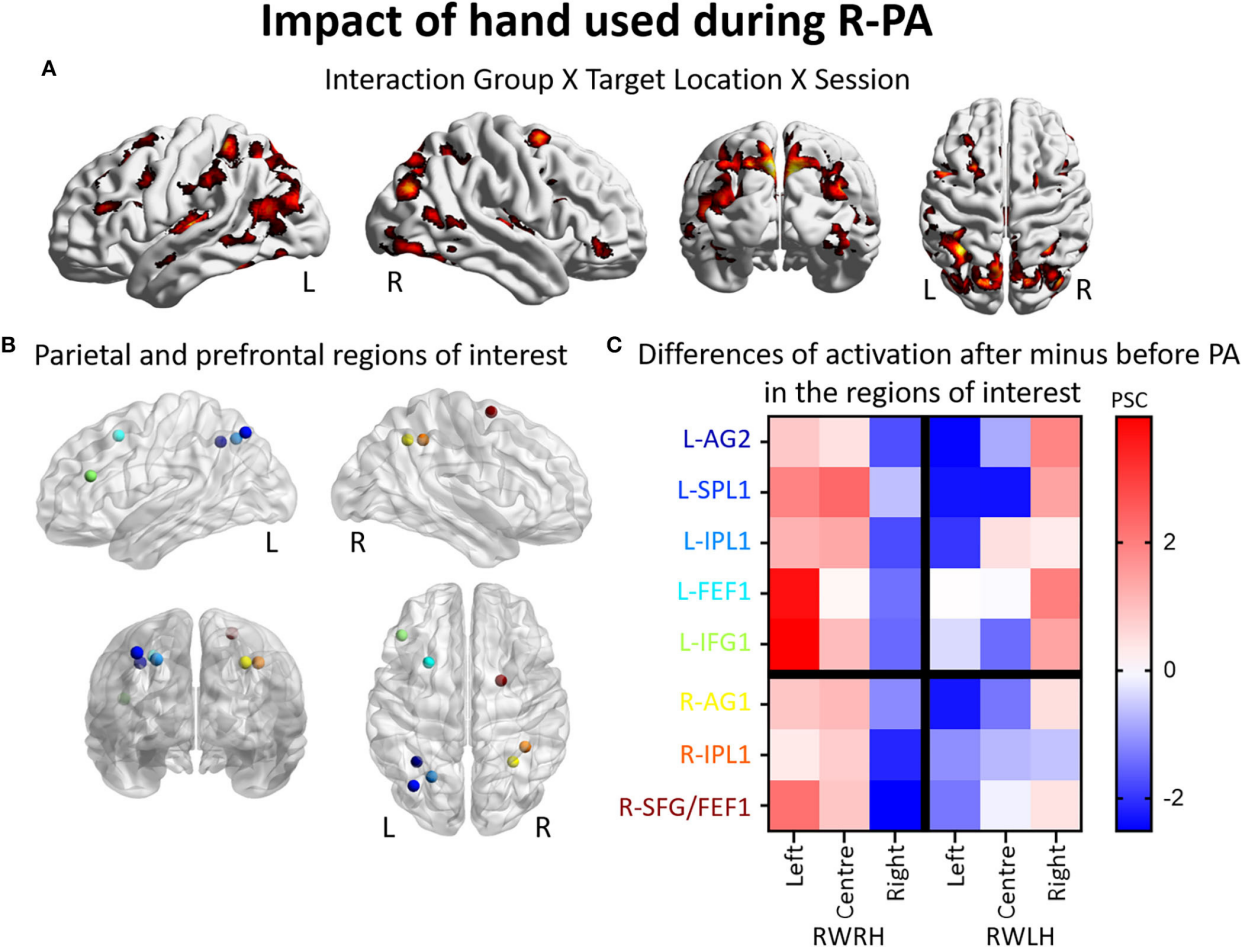
Impact of the hand used during R-PA on the reshaping of visuo-spatial representations. Modulation of brain activation as revealed by the ANOVA including the groups RWRH and RWLH. **(A**) Surface renderings of significant brain activation in the interaction between the factors Group (RWRH, RWLH) x Target location (left, center, right) x Session (pre, post), from left to right: lateral views of the left and right hemisphere, view from the back and the top. All maps are thresholded at *p* < 0.05, cluster extent k > 66. **(B)** Spheres located on the peaks of activation clusters in the parietal and prefrontal regions. Lateral views of the left and right hemispheres, above the back and top views. Color code denotes names of the regions, as indicated on the *y*-axis in C. **(C)** Differences of activation in percent signal change, after minus before PA, for each group (RWRH and RWLH) and each target position (left, center, and right). AG, angular gyrus; FEF, frontal eye field; IFG, inferior frontal gyrus; IPL, inferior parietal gyrus; L, left; PSC, percent signal change; R, right hemisphere; SFG, superior frontal gyrus; SMG, supramarginal gyrus; SPL, superior parietal lobule.

**Figure 3:**
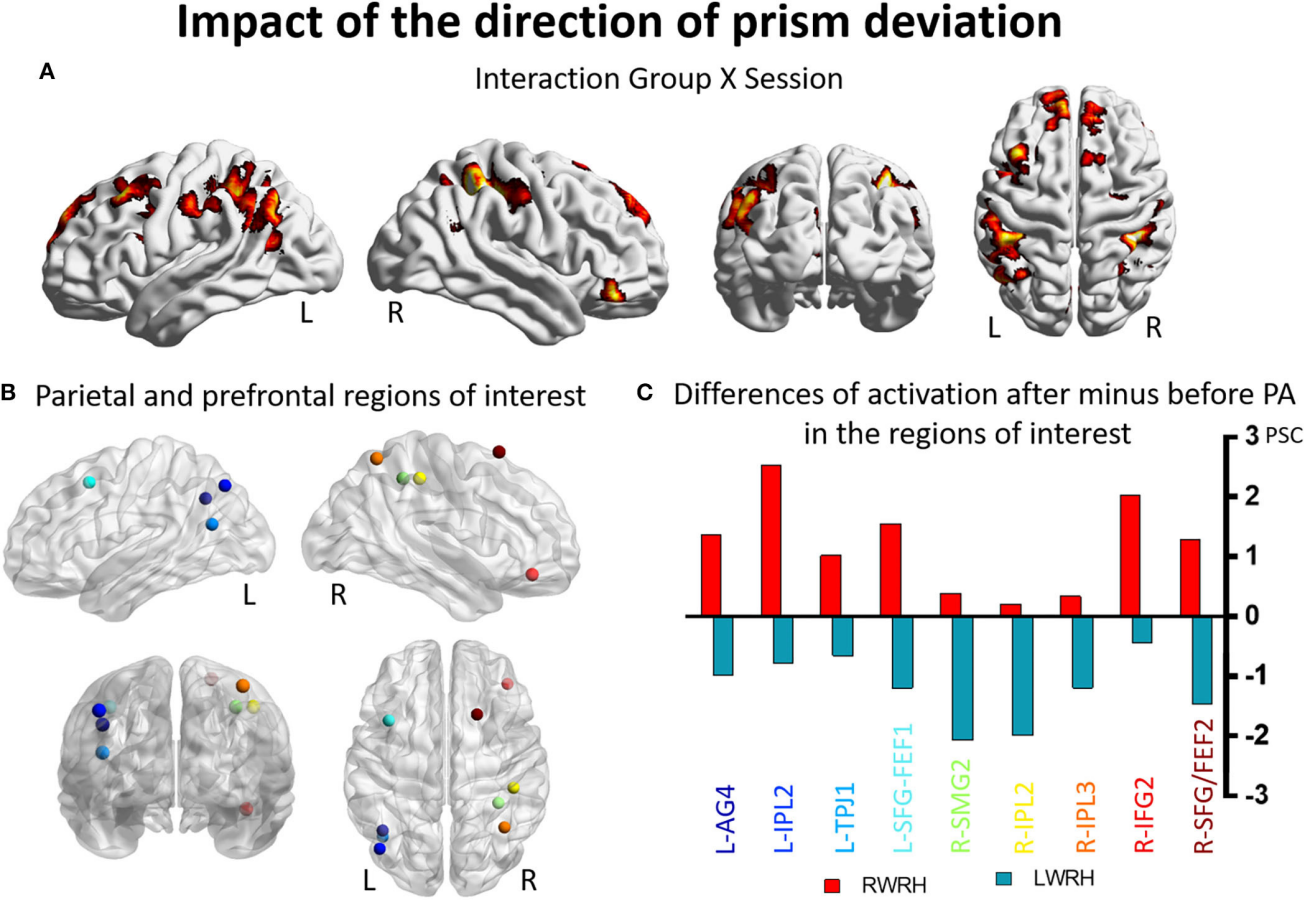
Impact of the direction of the prism deviation on the reshaping of visuo-spatial representations. Modulation of brain activation as revealed by the ANOVA including the groups RWRH and LWRH. **(A)** Surface renderings of significant brain activation in the interaction between the factors Group (RWRH, LWRH) x Session (pre, post), from left to right: lateral views of the left and right hemisphere, view from the back and the top. All maps are thresholded at *p* < 0.05, cluster extent k > 82. **(B)** Spheres located on the peaks of activation clusters in the parietal and prefrontal regions. Lateral views of the left and right hemispheres, above the back and top views. Color code denotes names of the regions, as indicated on the *x*-axis in C. **(C)** Differences of activation in percent signal change, after minus before PA, for the group RWRH (in red) and LWRH (in green). AG, angular gyrus; FEF, frontal eye field; IFG, inferior frontal gyrus; IPL, inferior parietal gyrus; L, left; PSC, percent signal change; R, right hemisphere; SFG, superior frontal gyrus; SMG, supramarginal gyrus; TPJ, temporo-parietal junction.

Activations are displayed in Figure 1A. Regions of interest (spheres in Figures 1–3) included in the listed clusters are indicated in parentheses. AG, angular gyrus; FEF, frontal eye field; IFG, inferior frontal gyrus; IPL, inferior parietal lobule; ITG, inferior temporal gyrus; MFG, middle frontal gyrus; MNI, Montreal Neurological Institute; MTG, middle temporal gyrus; SFG, superior frontal gyrus; SPL, superior parietal lobule; STG, superior temporal gyrus.

Thus, the three-way interaction Group (RWRH, RWLH, LWRH) x Session (pre *vs*. post) x Target location (left, center, right) reveals a set of IPL and prefrontal regions (Figure 1B, coordinates of these regions of interest in Table 3), where hand used during PA and the direction of prism deviation modulate differentially neural activity elicited by left, central and right visual targets (Figure 1C). Within left IPL clusters L-AG1 & L-AG2, left prefrontal cluster L-SFG/FEF1, right IPL cluster R-AG1, and right prefrontal clusters R-IFG1 and R-SFG/FEF1, the effect was driven by an increase in the neural activity elicited by the left and central targets (the latter except for L-SFG/FEF1) when right hand was used during R-PA. When the left hand was used, there was a tendency for an increase in the neural activity elicited by right targets. When the right hand was used during L-PA, there was a tendency in left IPL and prefrontal clusters to enhance activity elicited by the left and/or central targets (Figure 1C).

**Table 3:** Names and peak localisations of the parietal and prefrontal regions of interest extracted from the activations shown in Figure 1A (3 groups), Figure 2A (groups RWRH and RWLH), and Figure 3A (groups RWRH and LWRH) and used in the measures of percent signal changes shown in Figures 1C, 2C, 3C, respectively.

**3 Groups** **Interaction group x session x target location (see** **Figure 1****)**				**Groups RWRH and RWLH Interaction group x session x target location (see** **Figure 2****)**				**Groups RWRH and LWRH** **Interaction group x session (see** **Figure 3****)**			
**Name**	**MNI coordinates**			**Name**	**MNI coordinates**			**Name**	**MNI coordinates**		
L-AG1	−54	−64	36	L-AG2	−34	−52	42	L-AG4	−42	−58	36
L-AG2	−32	−52	44	L-SPL1	−36	−68	48	L-IPL2	−44	−70	44
L-AG3*	−46	−66	30	L-IPL1	−24	−62	44	L-TPJ1	−42	−62	20
L-SFG/FEF1	−24	16	42	L-FEF1	−26	14	46	L-SFG/FEF2	−38	14	46
R-SMG1*	60	−34	28	L-IGF1	−44	32	20	R-SMG2	34	−40	46
R-AG1	30	−52	42	R-AG1	30	−52	42	R-IPL2	44	−30	46
R-IFG1	46	38	−8	R-IPL1	38	−42	42	R-IPL3	38	−56	58
R-SFG/FEF1	24	4	60	R-SFG/FEF1	22	2	60	R-IFG2	40	38	−12
								R-SFG/FEF2	14	18	62

Asterisks mark two additional regions, which were identified in a previous study by contrasting the effect of R-PA to that of plain glasses (Crottaz-Herbette et al., 2014). AG, angular gyrus; FEF, frontal eye field; IFG, inferior frontal gyrus; IPL, inferior parietal lobule; L, left; MNI, Montreal Neurological Institute; R, right; SFG, superior frontal gyrus; SMG, supramarginal gyrus; TPJ, temporo-parietal junction.

#### Pattern of modulation in the inferior parietal lobule

The modulation of neural activity within IPL on either side was analyzed in individual significant peaks identified by the ANOVA Group x Target location x Session (Figure 1B, Table 3) and in locations identified in our previous study, which compared the effect of R-PA with that of pointing with plain glasses (Crottaz-Herbette et al., 2014).

In the left hemisphere, the present study identified two peaks in the angular gyrus (L-AG1 and L-AG2), whereas the previous study revealed an ROI near L-AG2 (designated L-AG3; Figures 1B,C, Table 3). In the right hemisphere, the present study identified one peak in the angular gyrus (R-AG1), whereas the previous study identified a nearby ROI on the posterior part of the supramarginal gyrus (R-SMG1; Figure 1B). For each of these five regions, the difference of activation between before *vs*. after prism adaptation is displayed separately for left, center, and right targets in Figure 1C.

In the RWRH group, there was a strong enhancement of activation elicited by left and central targets in the left IPL. In the right IPL, a decrease for right targets was observed (Figure 1C). This finding corresponds to the shift of activation between the left/right hemisphere and the left/right target described in our previous study (Crottaz-Herbette et al., 2014).

In the RWLH group, there was an increase in the activation elicited by left targets in L-AG3 and by right targets in all three IPL clusters on the left side. In R-AG1, a small increase in the right targets was observed.

In the LWRH group, there was a moderate decrease in the activation elicited by right targets in all the left IPL regions (L-AG1, L-AG2, L-AG3) and a large decrease in L-AG1 for left targets. On the right side, R-AG1 presented a moderate decrease for all targets.

Overall, RWRH and RWLH presented similar modulation in (left) AG3, with an increase for left and right targets. This modulation does not seem to depend on the hand used during the PA. This is not the case for L-AG1 and L-AG2, where the modulation is strikingly different between these two groups.

In summary, the detailed analysis of IPL confirms the previously reported enhancement of activation elicited by the left and central stimuli within the left IPL. This new analysis reveals that part of this enhancement is independent of the hand used for adaptation (in L-AG3, increased activation for left and right targets in the RWRH and RWLH groups), whereas in the other part of the IPL, the hand modulates the effect.

#### Impact of the hand used during R-PA on the reshaping of visuo-spatial representation

To determine the effect of the hand used during PA at the whole brain level, we compared activation patterns in the two groups, who were exposed to the same deviation, but who differed in hand used for adaptation. A mixed-design ANOVA with Group (RWRH, RWLH) as between-subject factor and Session (pre, post) and Target location (left, center, right) as within-subjects factors yielded significant interactions in several regions of the left and right IPLs (Figure 2A, Table 4). Additional clusters were found bilaterally in the lingual gyrus, precuneus, inferior and superior frontal gyrus, middle cingulate gyrus, middle temporal gyrus, and vermis 4 and 5 (cerebellum not shown in Figure 2). Additional activations were significant in the left middle frontal gyrus, the right inferior occipital gyrus, and middle temporal gyrus (Table 4).

**Table 4:** Brain regions showing significant effects in the interaction between the factors group x session x target location, in the three-way ANOVA including the 2 groups (RWRH, RWLH).

**2 groups: RWRH RWLH**	**N of**	**Peak**	**Peak MNI** **coordinates**		
**Areas**	**voxels**	**intensity**	**x**	**y**	**z**
Bilateral precuneus, SPL, AG, cingulate gyrus; Left fusiform and postcentral gyri, ITG, MTG, STG; Right superior and middle occipital gyri (including L-AG2, L-SPL1, L-IPL1, R-AG1, R-IPL1)	6,394	12.69	14	−66	36
Left insula, STG, transverse temporal, precentral and postcentral gyri	609	11.79	−46	−18	10
Left MFG, SFG, FEF (including L-FEF1)	519	10.00	−26	14	46
Left caudate, limbic lobe, anterior cingulate gyrus	275	9.87	−16	22	−4
Right declive of vermis, vermis 4, cerebellum 6	185	9.68	12	−68	−26
Left MTG, STG	114	8.92	−48	0	−10
Right cingulate gyrus, posterior cingulate	215	8.53	2	−24	28
Right caudate	72	8.43	18	2	22
Right MFG, SFG (including R-SFG/FEF1)	255	8.40	26	2	60
Right MTG, inferior, middle and occipital gyri, STG, ITG	889	7.60	48	−54	10
Left MFG, IFG (including L-IGF1)	89	7.22	−44	32	20
Right IFG, MFG	129	6.92	46	38	−8
Left IFG, MFG	419	6.85	−48	6	28
Right MFG, IFG	199	6.66	44	0	42
Left posterior cingulate gyrus, anterior cerebellum lobe	169	6.22	−12	−56	6
Left MTG, STG	149	5.78	−66	−34	4
Left IPL, postcentral and precentral gyri	186	5.22	−62	−28	36
Left middle cingulate and medial frontal gyri, SFG	155	5.10	−2	24	54
Right SFG, MFG	74	5.02	22	12	46
Right calcarine sulcus, posterior cingulate gyrus	90	4.66	22	−58	18

Activations are displayed in Figure 2A. Regions of interest (spheres in Figures 1–3) included in the listed clusters are indicated in parentheses. AG, angular gyrus; FEF, frontal eye field; IFG, inferior frontal gyrus; IPL, inferior parietal lobule; ITG, inferior temporal gyrus; MFG, middle frontal gyrus; MNI, Montreal Neurological Institute; MTG, middle temporal gyrus; SFG, superior frontal gyrus; SPL, superior parietal lobule; STG, superior temporal gyrus.

Several clusters of interest were identified by the three-way interaction Group (RWRH, RWLH) x Session (pre, post) x Target location (left, center, right). Within IPL, these clusters were located in the superior-medial part (Figures 2A,B, coordinates of these regions of interest in Table 3). Within the prefrontal convexity, they comprised the frontal eye field and the inferior frontal gyrus (Figures 2A,B). Within IPL and prefrontal clusters, both in the right and left hemispheres, the interaction was driven by an increase in neural activity elicited by left and central targets and a decrease in neural activity elicited by right targets, when the right hand was used during R-PA (Figure 2C). When the left hand was used during R-PA, neural activity elicited by left targets tended to decrease (Figure 2C).

#### Impact of the direction of prism deviation on the reshaping of visuo-spatial representation

To determine the effect of the direction of prism deviation at the whole brain level, we compared activation patterns in the two groups, who were exposed to opposite deviations but used the right hand for adaptation.

A mixed-design ANOVA with Group (RWRH, LWRH) as between-subject factor and Session (Pre, Post) and Target location (left, center, right) was conducted. In this ANOVA, the three-way interaction including the factors Group (RWRH, LWRH) x Session (pre, post) x Target location (left, center, right) did not yield significant activation in attentional regions; only small clusters with very low activation were observed. Further analysis of this ANOVA was done with the Group x Session interaction (Figure 3A). Large clusters were present bilaterally in the IPL (including the left angular, and the left and right supramarginal gyri), postcentral gyrus, inferior and superior frontal gyri, as well as in the right superior temporal gyrus, the left middle frontal, and superior parietal gyri (Figure 3, Tables 3, 5).

**Table 5:** Brain regions showing significant effects in the interaction between the factors group x session x target location, in the three-way ANOVA including the 2 groups (RWRH, LWRH).

**3 groups: RWRH LWRH**	**N of**	**Peak**	**Peak MNI** **coordinates**		
**Areas**	**voxels**	**intensity**	**x**	**y**	**z**
Right SMG, IPL, SPL, postcentral and precentral gyri (including R-SMG2, R-IPL2, R-IPL3)	1,053	21.05	34	−40	46
Right IFG, MFG (including R-IFG2)	207	17.42	40	38	−12
Left postcentral gyrus, IPL,SMG, SPL, AG, STG, MTG, TPJ (including L-AG4, L-IPL2, L-TPJ1)	1,940	12.12	−46	−34	50
Left SFG, MFG	501	11.78	−6	58	36
Left precentral gyrus, IFG, MFG	278	11.08	−34	4	36
Left MFG, SFG (including L-SFG/FEF2)	374	10.94	−38	14	46
Right SFG, medial frontal gyrus (including R-SFG/FEF2)	526	10.68	12	48	42
Left posterior cingulate and lingual gyri, cuneus, precuneus	294	10.36	−12	−60	6
Right AG, IPL	110	9.37	52	−50	28
Right MFG, precentral gyrus	93	6.71	24	−8	52

Activations are displayed in Figure 3A. Regions of interest (spheres in Figures 1–3) included in the listed clusters are indicated in parentheses. AG, angular gyrus; FEF, frontal eye field; IFG, inferior frontal gyrus; IPL, inferior parietal lobule; MFG, middle frontal gyrus; MNI, Montreal Neurological Institute; MTG, middle temporal gyrus; SFG, superior frontal gyrus; SMG, supramarginal gyrus; SPL, superior parietal lobule; STG, superior temporal gyrus; TPJ, temporo-parietal junction.

Several clusters identified by the interaction Group (RWRH, LWRH) x Session (pre, post) were within regions known to be involved in attention. Within left IPL these clusters were located on the angular gyrus and in the anterior part of IPL, within right IPL in its anterior and superior parts (Figures 3A,B). Within the prefrontal convexity, they comprised the frontal eye field and the inferior frontal gyrus (Figures 3A,B). Within the left IPL and prefrontal clusters and within the right prefrontal clusters the interaction was driven by an increase in neural activity elicited by visual targets when right-deviating prisms were used and a decrease, when left-deviating prisms were used (Figure 3C). Within the right IPL, the interaction was driven by a decrease in neural activity elicited by visual targets when left-deviating prisms were used (Figure 3C, coordinates of these regions of interest in Table 3).

## Discussion

The present study confirms the previously described enhancement of left and central visual field representation within the left IPL following brief exposure to R-PA (i.e., Crottaz-Herbette et al., 2014). It further shows that the use of right *vs*. left hand during adaptation modulates this enhancement in some but not all parts of left IPL. Interestingly, the right hand used in L-PA mimics in parts the effect of R-PA by enhancing activation elicited by left stimuli in left IPL and by decreasing activation elicited by right stimuli in right IPL.

### Impact of the hand used during R-PA on the reshaping of the visuo-spatial representation

At the behavioral level, the combination of hand and direction of prism deviation impacted the extent of the direct sensorimotor after-effect, which was larger when the right hand was used during the R-PA. It is to be noted that the after-effect was measured with the hand used for adaptation. The effect of the use of right *vs*. left hand in PA has been reported previously, but without addressing the issue systematically.

Two previous studies used alternately either hand for pointing during adaptation, with switches between left and right hand either every minute (Michel et al., 2008) or every 25 pointings (Reed and Dassonville, 2014). Both of these studies did not find significant differences between the after-effects after R-PA and L-PA, using an open-loop manual point task (Michel et al., 2008) or a subjective straight-ahead task (Reed and Dassonville, 2014). However, both of these studies used the right hand in the tasks measuring the after-effects (more precisely, the dominant hand in Reed and Dassonville's study). In another study, the comparison of normal subjects, who underwent either R-PA with the right hand or L-PA with the left hand, demonstrated a larger after-effect in a proprioceptive task in the former, but the difference was not tested statistically (Ronchi et al., 2019).

Our analysis of activation patterns shows that the use of right *vs*. left hand during R-PA modulates the reshaping of left IPL. The enhancement of left space representation induced by R-PA involves a smaller part when the left hand is used (i.e., increase of activation only in L-AG3 for the RWLH group, as shown in Figure 2). As proposed previously, the hand used during R-PA may modulate hemispheric imbalance, which is induced by the adaptation (Michel et al., 2008). Putative mechanisms are believed to involve visuomotor recalibration and spatial realignment occurring during the adaptation phase. Several studies investigated these phases with fMRI, but did not compare directly the effect of hand.

The use of the right hand in R-PA was shown to activate the primary motor cortex and anterior intraparietal sulcus, medial cerebellum, and anterior cingulate on the left side during the early phase of adaptation (Danckert et al., 2008). A later study, also using L-PA with the right hand, showed increased activation in the left and right superior temporal sulci and gyri, intraparietal sulcus and IPL, and the right cerebellum, with specific timing for these regions during the deployment of adaptation (Luauté et al., 2009). During L-PA with the right hand, Chapman and colleagues (Chapman et al., 2010) showed increased activation in the cerebellum and IPL, during the early phase of the adaptation. Altogether, these studies reported bilateral changes of activation during PA with the right hand mainly in the parietal, temporal, and cerebellar regions but did not show a clear distinction between L-PA and R-PA.

During R-PA, the subjects need to learn to point to the left to reach the targets, which they perceive at the fixation point. Pointing with the right hand implies reaching on the side of the fixation point opposite to the hand, whereas pointing with the left hand, reaching on the same side. This situation may impact the recalibration and spatial realignment. Pointing (without prisms) has been shown to involve bilaterally the intraparietal sulcus; this region is more activated for contra- than ipsilateral targets and this activity is modulated by the hand which is used for pointing. The activation is stronger when the contralateral hand reaches for contralateral targets than the contralateral hand for ipsilateral targets (Medendorp et al., 2005).

L-PA and R-PA have been shown to alter exploratory eye movements, introducing a shift in the same direction as the adaptation after-effect (Ferber and Murray, 2005; Bultitude et al., 2013). Whether exploratory eye movements are also modulated by the hand used during PA is currently unknown. Two observations suggest that it may be so and speak for further investigations. As shown in this study, the R-PA-induced after-effect is modulated by the hand used during PA. Depending on which hand is used during PA may also affect eye exploration as well. Putative neural mechanisms need to be explored, but parts of the posterior parietal cortex, known to support both reaching and saccades toward visual targets (Beurze et al., 2009) may be involved.

### Impact of the direction of the prism deviation on the reshaping of visuo-spatial representation

In clusters that were identified in the IPL with the three-way interaction Group x Target location x Session, we have seen only a small effect of adaptation in the LWRH group (Figure 1). In the left IPL, the use of the right hand in L-PA mimics in parts the effect of R-PA by enhancing activation related to left stimuli and in the right IPL by decreasing the activation related to right stimuli.

In a series of studies (Crottaz-Herbette et al., 2014, 2017a,b, 2019), we showed that L-PA and R-PA have the opposite effect on the right hemispheric dominance for attention, in healthy individuals and patients with unilateral brain lesions. R-PA with the right hand decreases the right hemispheric dominance for attentional processing, while L-PA with the left hand enhances this dominance. In this study, the modulation within the right SMG remains the same whether a prism adaptation with the right hand uses rightward or leftward prisms (i.e., RWRH and LWRH groups). In both cases, there is a decrease in the activation in the right SMG, larger for right, than for left, stimuli. This was not the case in our previous study (Crottaz-Herbette et al., 2017b) using L-PA and left hand, where we showed an increased activation only in the right angular gyrus for right targets, and no decrease. This apparent discrepancy is because the clusters analyzed in this study and those analyzed in the previous study on L-PA (Crottaz-Herbette et al., 2017b) are not in the same locations, which were identified on the basis of different hypotheses with different experimental designs.

The effect of L-PA was also investigated by Martin-Arevalo and colleagues (Martín-Arévalo et al., 2016) using the Posner task and event-related potentials (ERPs). ERPs in responses to left, as compared to right cues, were found to be smaller, reflecting an orienting bias toward rightward cues following L-PA. Furthermore, smaller ERPs were found for the invalidly cued left than right targets, interpreted as disengagement deficit from the right space. These results were interpreted as bilateral modulations of the dorsal attentional network (DAN), highlighting the role of the interhemispheric connections as well as the interaction with the cerebellum.

### Heterogeneity of the inferior parietal lobule

IPL is classically subdivided into two parts, the angular gyrus in its posterior and the supramarginal gyrus in its anterior part (Seghier, 2013). Cytoarchitectonically it consists of seven areas, five of which are within the supramarginal and two within the angular gyrus (Caspers et al., 2006); the former are referred to as PFop, PFt, PFm, and PFcm and the latter as PGa and PGp [PF and PG following the cytoarchitectonic designation by von Economo and Koskinas (1925)]. The different parts of IPL differ in their respective densities of different transmitter receptors (Caspers et al., 2013). Integrating cytoarchitectonic, connectional, and functional information, Caspers et al. (2012) regrouped these seven regions into three different clusters, a rostroventral (areas PFt, PFop, and PFcm), an intermediate (areas PF and PFm), and a caudal group (areas PGa and PGp).

**Figure 4:**
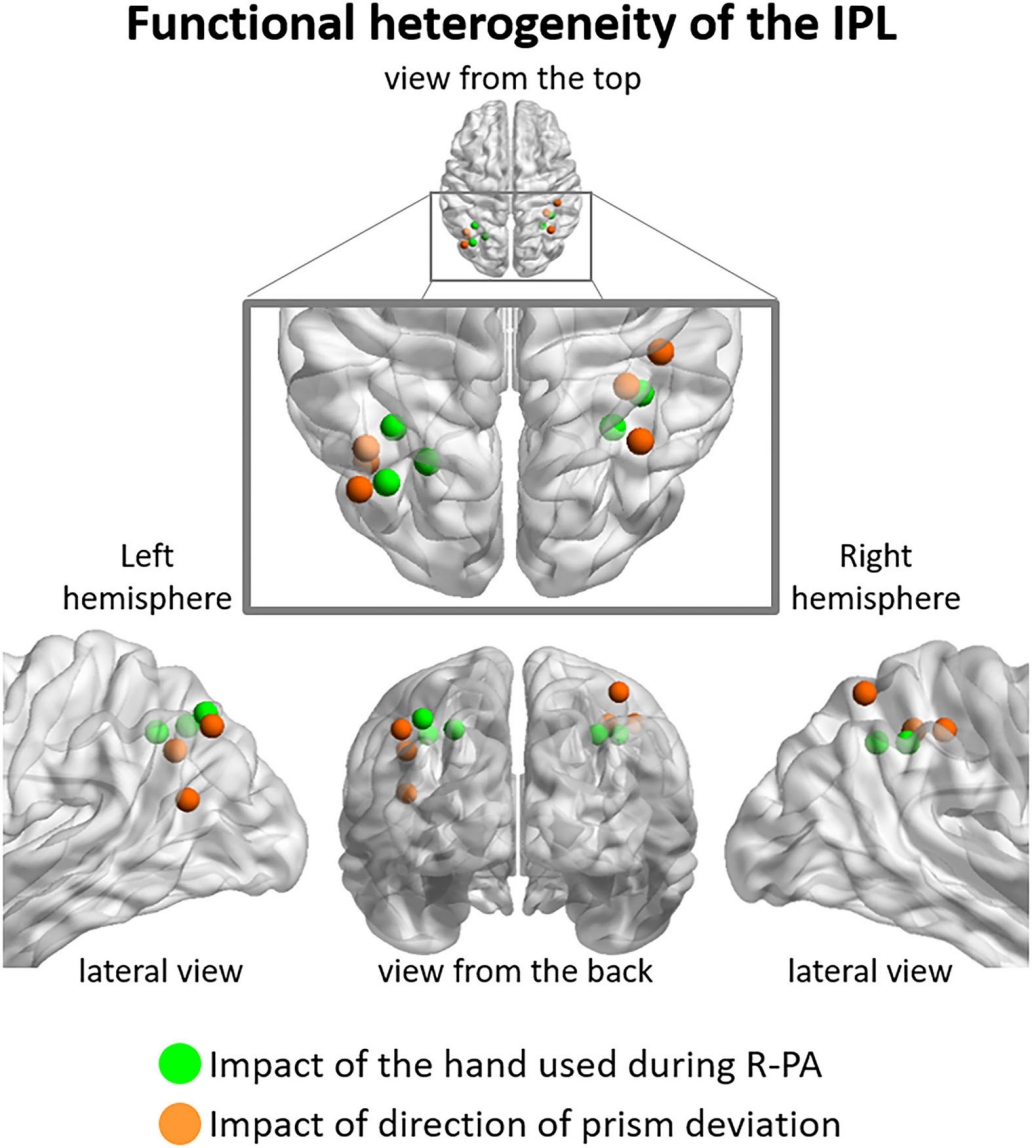
Functional heterogeneity of IPL, as revealed by the impact used during R-PA *vs*. (green dots), the direction of prism deviation (orange dots). The former correspond to peaks of activity determined by three-way ANOVA Group (RWRH, RWLH) x Target location (left, center, right) x Session (pre, post) illustrated in Figure 2, the latter to those of two-way ANOVA Group (RWRH, LWRH) x Session (pre, post) illustrated in Figure 3. Note that in the left hemisphere regions impacted by the hand used during R-PA are located in the supero-medial part of IPL and those impacted by the direction of the prism deviation in the latero-posterior part. Less clear segregation is present in the right hemisphere.

The above described anatomical heterogeneity is compatible with our observation that distinct parts of IPL are differentially modulated by the direction of prism orientation and/or the hand used during PA. Within left IPL, the hand used during R-PA modulated neural activity elicited within the supero-medial part of the angular and the posterior part of the supramarginal gyrus (Figures 3A, 4). The use of the right hand during R-PA enhanced the neural activity elicited by left targets, whereas the use of the left hand enhanced the neural activity elicited by right targets (Figure 2C). The direction of prism deviation modulated neural activity within the lateral and inferior part of the angular gyrus, where neural responses to visual targets were enhanced by R-PA and decreased by L-PA [Figures 3, 4; (Crottaz-Herbette et al., 2014)]. Within right IPL, the hand used during R-PA modulated neural activity within the supero-medial part of the angular and supramarginal gyri and the direction of prism deviation within the superior part of the angular gyrus (Figure 4). The use of the right hand during R-PA enhanced neural activity elicited by left targets, whereas the use of the left hand had only a very marginal effect (Figures 1, 2). The use of right-deviating prisms had little effect within the superior part of the angular gyrus, whereas the use of left-deviating prisms decreased the activity elicited by visual targets (Figure 3). The part of right IPL, which has been reported in a previous study to enhance its responsiveness to right visual targets following L-PA is located in the postero-inferior part of the angular gyrus, below the foci identified in the present study (Crottaz-Herbette et al., 2017b). This former study tested a different hypothesis, from the one addressed here. It compared the effect of L-PA, R-PA, and that of plain glasses using a whole brain analysis with a three-way ANOVA Group (L-PA, R-PA, plain glasses) x Session (pre *vs*. post) x Target location (left, center, right). A region within the infero-posterior part of right IPL yielded a significant three-way interaction, driven by the increase in responses to right targets after L-PA.

Thus, our results indicate that the antero-superior part of IPL is modulated by the hand used during R-PA and the posterior and inferior parts by the direction of prism deviation. This apparent functional dichotomy concurs with prior reports. The anterior part of IPL has been associated with reaching and grasping tasks, with the usage of tools or the imitation of its usage (Peeters et al., 2009). The intermediate region is active in spatial attention and reorienting tasks (Rushworth et al., 2001; Corbetta et al., 2008).

### Modulations beyond the VAN

Finally, our results show that hand modulates the effect of R-PA in several regions which are part of the attentional system and to an extent beyond the IPL (Figure 3A). Bilateral regions of the DAN (superior frontal gyri and superior parietal gyri, Figure 3) also showed modulation after R-PA. These regions of the DAN were also modulated after R-PA in a previous fMRI study (Saj et al., 2013) using tasks that rely on the DAN. In patients with right hemispheric damages, they showed increased activation during a visual search and line bisection tasks in the superior parietal lobule, superior frontal gyrus, and lateral occiptal cortex, in the left hemisphere, and also in the right hemisphere when the regions were spared. The visual target detection task used in this study is known to activate preferably the VAN, however, because of the strong links between the VAN and the DAN (Corbetta and Shulman, 2002), functional changes after R-PA in the VAN might also have a functional impact on the DAN.

Our study aimed to determine whether visuo-spatial representations within the attentional system are modulated by PA and/or by the hand used during PA. In other words, are there changes in neural populations, which encode a specific stimulus (here visual targets presented at left, central, or right locations). For this, the task needed to be equally well performed before *vs*. after PA, which was the case for target detection in this study and in previous studies (Crottaz-Herbette et al., 2014; Tissieres et al., 2018). Another approach to investigate PA-induced modulation of VAN and beyond would be to explore neural correlates of task difficulty in normal subjects or during recovery in patients. For this task to be selected, the performance in which it is modulated by PA, such as line bisection by L-PA.

### Limitations of the study

First, eye movements during the visual detection task were not measured. Visual targets were large bright white stars on a black background and were easily detected without eye movements. We cannot, however, exclude that participants performed eye movements during the task and that such eye movements might be driven by changes in brain activation. Using the eye tracking system during fMRI acquisition in further studies will help to specify how eye movements influenced the brain modulations reported after PA. Second, the visual detection task used during fMRI acquisition is a task involving the ventral stream of attention. It would be interesting to compare the modulation after PA in tasks involving more of the dorsal stream. Finally, we did not monitor the time course of the after-effect of the R-PA and L-PA. A different time course for the after-effects following R-PA *vs*. L-PA would provide valuable information about the mechanisms underlying these adaptations. These limitations should be addressed in further studies using eye tracking during fMRI acquisitions, additional tasks involving the dorsal stream of attention, and by conducting repeated measures of the pointing errors after PA.

## Conclusion

Our results show that the use of the right *vs*. left hand modulates the reshaping of the attentional system. This calls for more investigations on the interactions between prism adaptation and the hand used. Two approaches may be particularly worthwhile. First, the effects of R-PA trained with the right or the left hand should be explored in more detail. In addition to target detection, which we used in this study, tasks known to depend on the DAN should be investigated. Of particular interest would be the perceptual and motor versions of line bisection. These results would be of conceptual interest but could also have an impact on clinical applications of R-PA. Second, besides neglect, R-PA was shown to alleviate clinical conditions such as complex regional pain syndromes (Sumitani et al., 2007; Bultitude and Rafal, 2010; Foncelle et al., 2021). The combination of hand used for adaptation and direction of prism deviation might be worth exploring in these conditions where motor function tends to be preserved on both sides.

## Data availability statement

The raw data supporting the conclusions of this article will be made available by the authors, without undue reservation.

## Ethics statement

The studies involving human participants were reviewed and approved by Commission cantonale d'éthique de la recherche sur l'être humain du Canton de Vaud, Suisse. The patients/participants provided their written informed consent to participate in this study.

## Author contributions

NF contributed to the data collection, data processing, statistical analyses, and manuscript preparation. SC contributed to the study design, data analyses, and manuscript preparation. SC-H contributed to the study design, data collection, data analyses, and manuscript preparation. All authors contributed to manuscript preparation and approved the submitted version.

## Funding

This work was supported by the Swiss National Science Foundation (http://www.snf.ch) grants to SC (FNS 320030-159708) and SC-H (Marie-Heim-Vögtlin fellowship FNS PMPDP3_129028), and by the Gianni Biaggi de Blasys Foundation to SC-H. Open access funding provided by University of Lausanne.

## Conflict of interest

The authors declare that the research was conducted in the absence of any commercial or financial relationships that could be construed as a potential conflict of interest.

## Publisher's note

All claims expressed in this article are solely those of the authors and do not necessarily represent those of their affiliated organizations, or those of the publisher, the editors and the reviewers. Any product that may be evaluated in this article, or claim that may be made by its manufacturer, is not guaranteed or endorsed by the publisher.
